# Acute and chronic infections with nonprimate hepacivirus in young horses

**DOI:** 10.1186/s13567-016-0381-6

**Published:** 2016-09-22

**Authors:** Theresa Gather, Stephanie Walter, Stephanie Pfaender, Daniel Todt, Karsten Feige, Eike Steinmann, Jessika M. V. Cavalleri

**Affiliations:** 1 Clinic for Horses, University of Veterinary Medicine Hannover, Foundation, Bünteweg 9, 30559 Hannover, Germany; 2 Institute for Experimental Virology, TWINCORE Centre for Experimental and Clinical Infection Research, Feodor-Lynen-Str. 7, 30625 Hannover, Germany

## Abstract

The recently discovered nonprimate hepacivirus (NPHV) naturally infects horses and is the closest known homolog of hepatitis C virus to date. Within a follow-up study acute field infections were monitored in four young Thoroughbred horses until the ages of 12–13 months. Serum samples were analyzed for the presence of NPHV RNA and anti-NPHV NS3 antibodies and liver specific parameters were evaluated. The four young horses were not able to clear infection, but remained chronically infected for the entire monitored time period despite the presence of NPHV specific antibodies.

## Introduction

Nonprimate hepacivirus (NPHV) was described to infect horses in several countries worldwide and represents a new member within the family *Flaviviridae*, genus *Hepacivirus* [1, 2]. Among all hepaciviruses, NPHV is the closest known homolog of hepatitis C virus (HCV) to date. HCV is a major human pathogen with approximately 146 million people infected worldwide [3]. In 75–85% of acutely infected patients progression to chronic disease occurs, which can lead to hepatic fibrosis, cirrhosis and carcinoma [4]. In recent years, similar as well as distinct features between HCV and NPHV have been described. NPHV is highly prevalent in horses with 30–40% seropositivity and 3% viremia [1, 5–10]. However, disease association remains uncertain although several studies reported subclinical hepatitis in infected horses. A mild elevation of serum liver enzymes was observed at seroconversion in some affected horses, although in most horses serum liver enzymes remained within the reference range [7, 11, 12]. Single reports exist about NPHV infected horses suffering from severe hepatitis. Yet, a causative relationship still needs to be confirmed [12, 13]. Comparable to HCV infection, hepatotropism has been confirmed for NPHV [11, 14]. Nevertheless, the routes of NPHV transmission remain mostly unclear. Similar to HCV a parenteral infection route via direct blood–blood contact has been described in horses by experimental inoculation with infectious plasma containing NPHV [12]. In a recent study, we investigated twenty mare-foal pairs at parturition until 6 months postpartum and could show the occurrence of vertical transmission of NPHV at parturition [15]. Moreover, we observed transmission of NPHV isolates within the respective pasture herds indicating the possibility of a direct transmission between horses. Interestingly, we could show that several foals became viremic within the first 6 months of life [15]. To shed some light on the course of naturally acquired NPHV infection of the young horses, we aimed to perform a follow-up study by monitoring four foals (foal # 9, # 10, # 17 and # 19) until the age of 12–13 months.

## Materials, methods and results

**Figure 1:**
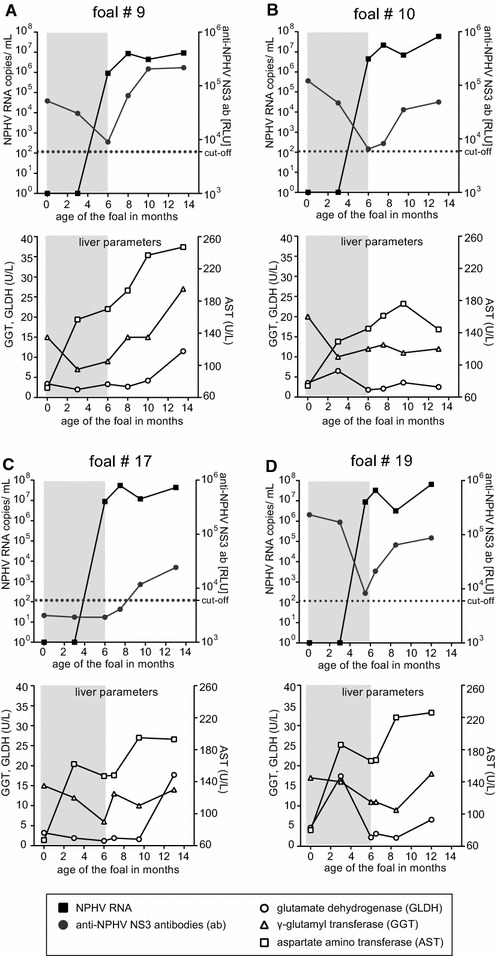
**Course of infection in the four foals # 9, # 10, # 17 and # 19 from parturition until the ages of approximately 13** **months.** Serum samples from the first three sampling time points (at parturition and at the ages of 3 and 6 months are derived from a previous study (marked with a grey background) [15]. Until the ages of 12–13 months these foals were further monitored within this follow-up study and serum was taken at three additional time points. All serum samples were analyzed for the presence of anti-NPHV NS3 antibodies (ab) (*grey bullet*) and NPHV RNA (*black square*) by LIPS and qRT-PCR, respectively. The cut-off limit for the LIPS was determined by the mean value of wells containing only buffer A, the RUC-NS3 fusion protein and A/G beads plus three standard deviations and is illustrated as dashed line. In the lower panels, liver specific parameters (GLDH, GGT and AST) are shown for each foal at the three follow-up time points as unfilled symbols, with the following reference ranges set by the laboratory: GLDH < 6 U/L, GGT < 20 U/L and AST < 170 U/L. (**A**) Foal # 9, (**B**) foal # 10, (**C**) foal # 17 and (**D**) foal # 19.

Blood samples were taken from these four foals at indicated time points (Figure 1; Table 1). All samples were obtained with full owner consent as part of routine health management. Additionally, a general examination of the foals was conducted at all investigated time points. Next, sera was analyzed for the presence of NPHV RNA and anti-NPHV NS3 antibodies by a SYBR Green based quantitative real-time PCR (qRT-PCR) and luciferase immunoprecipitation assay (LIPS), respectively, as described earlier [1, 11, 15]. As shown in Figure 1, NPHV RNA was detected in the serum of all foals 6 months after birth. From the initial virus detection onwards the four acutely infected young horses remained viremic with high viral loads during the entire monitored 7 months and none of them eliminated the virus until the last sampling at the age of 12–13 months (Figure 1A–D). For the foals # 9, # 10 and # 19 anti-NPHV NS3 antibodies were detected at the time point of birth, decreased until 6 months postpartum below or close to the cut-off of the assay and NPHV specific antibodies appeared again after NPHV RNA was detected in the serum (Figure 1A, B and D). Of note, foal # 17 was the only foal not receiving maternal anti-NPHV NS3 antibodies after foaling and became newly infected at a similar age as the remaining foals and production of anti-NPHV NS3 antibodies was observed from the age of 10 months onwards (Figure 1C). In Table 1 details on the NPHV RNA and anti-NPHV NS3 antibody status of the follow-up samples of the four horses are given (Table 1).

**Table 1:** **Raw values of NPHV RNA and anti-NPHV NS3 antibodies of the acutely infected foals**

Follow-ups samples			
	Sample # I	Sample # II	Sample # III
Foal # 9			
Age in months	8	10	13.5
Anti-NPHV NS3 ab (RLU)	*6.57E* *+* *04*	*2.06E* *+* *05*	*2.18E* *+* *05*
NPHV RNA (copies/mL)	8.67E + 06	4.45E + 06	9.27E + 06
Foal # 10			
Age in months	7.5	9.5	13
Anti-NPHV NS3 ab (RLU)	*8.19E* *+* *03*	*3.50E* *+* *04*	*4.86E* *+* *04*
NPHV RNA (copies/mL)	2.15E + 07	6.88E + 06	5.81E + 07
Foal # 17			
Age in months	7	9.5	13
Anti- PHV NS3 ab (RLU)	4.07E + 03	*1.17E* *+* *04*	*2.43E* *+* *04*
NPHV RNA (copies/mL)	5.44E + 07	1.20E + 07	4.36E + 07
Foal # 19			
Age in months	6.5	8.5	12
Anti-NPHV NS3 ab (RLU)	*2.12E* *+* *04*	*6.45E* *+* *04*	*8.68E* *+* *04*
NPHV RNA (copies/mL)	3.25E + 07	3.17E + 06	6.44E + 07

All collected follow-up serum samples were analyzed for the presence of NPHV RNA and anti-NPHV NS3 antibodies (ab) by qRT-PCR and LIPS, respectively. NPHV RNA titers are displayed as RNA copies/mL. Anti-NPHV NS3 ab values are given as RLU, whereas values above the cut-off are highlighted in italic numbers.

To investigate the clinical relevance of NPHV infection for liver disease, the horses were clinically examined and blood was analyzed for the liver specific enzymes glutamate dehydrogenase (GLDH), γ-glutamyl-transferase (GGT) and aspartate aminotransferase (AST) in the laboratory of the small animal clinic at the University of Veterinary Medicine Hannover, Foundation. Results for each sampling time point are shown in Figure 1, lower panels. A mild elevation of liver enzymes was observed at the age of 12–13 months for foals # 9, # 17, and # 19. However, values of all other time points remained within the reference range and no clinical signs indicating liver disease were found at any sampling time point.

In conclusion, four acutely infected foals developed chronic infection and remained viremic with high viral loads during the entire monitored 7 months. Despite the presence of anti-NPHV NS3 antibodies, the four young horses were not able to clear the infection within the monitored time period.

## Discussion

This study provides the first description of the clinical course of naturally acquired NPHV infection in young horses. This follow-up study was conducted in the context of a previous study investigating the occurrence of vertical transmission of NPHV in a horse cohort, where we monitored twenty Thoroughbred broodmares and their foals from parturition until 6 months after foaling [15]. Here, we monitored four of these foals for another 7 months. These foals became NPHV RNA positive at the age of 6 months and stayed viremic for another 7 months with the concurrent detection of anti-NPHV NS3 antibodies. The activity of liver specific enzymes increased in three foals at the ages of 12–13 months. The mild elevation of enzyme activity could indicate a subclinical hepatitis due to the persistent NPHV infection. For reliable diagnosis liver biopsies would be required, since the elevation of liver enzymes might have had an unrelated reason.

In a retrospective study Matsuu et al. also detected NPHV infections in 5 out of 7 young horses aged between 4 and 6 months and 2 years [16], supporting the time of infection in our foals. In three foals (foal # 9, # 10, and # 19) maternal anti-NPHV NS3 antibodies were detected after birth until three to 6 months postpartum (Figures 1A, B and D). One could speculate that after degradation of maternal antibodies, the foals are more susceptible to a NPHV infection. However, foal # 17 did not receive protection by maternal antibodies, yet it became infected at the same age as the three other foals. Moreover, it is not clear whether the maternal antibodies protect against a NPHV infection. So far, the course of infection for NPHV in young horses has only been described by Ramsay et al. who experimentally infected two foals at the age of 2–4 weeks and monitored them for more than 1 year post-infection [12]. These foals became viremic shortly after inoculation and remained persistently infected for the entire monitored 63 weeks. Anti-NPHV antibodies were detected from the age of approximately 8 weeks onwards and peaked 23 weeks post-infection. In the following weeks, the detection of antibodies decreased in parallel with the viral loads that sharply declined from 40 weeks post-infection onwards. Interestingly, in our study four naturally infected foals also remained persistently infected during the entire monitored time with high viral loads and were not able to clear the infection despite the presence of antibodies from the age of 7 to 9 months onwards. The reason for the inability of the four young horses to eliminate NPHV remains unclear. The development of the immune system of young horses is only partially understood. It is known, that the onset of the adaptive immune response is delayed and antibody responses of type IgG4, IgG7 and IgE as well as T cell and cytokine responses slowly evolve within the first year of life [17]. However, to understand the complex interaction of NPHV and the equine immune system, further research is required. It has been shown, that adult horses are often able to clear NPHV infection within 2 months after acute infection although persistently infected horses have also been described analogous to chronic HCV infection in humans [11, 12].

In conclusion, in four young horses the NPHV field infection progressed from acute to chronic infection. The four horses were not able to clear NPHV until the ages of 12–13 months despite the presence of antibodies for several months.

## Abbreviations

AST: aspartate amino transferase
GGT: γ-glutamyl transferase
GLDH: glutamate dehydrogenase
HCV: hepatitis C virus
LIPS: luciferase immunoprecipitation system
NPHV: nonprimate hepacivirus
NS 3: nonstructural protein 3
RLU: relative light units
qRT-PCR: quantitative real-time polymerase chain reaction

## References

[1] Burbelo PD, Dubovi EJ, Simmonds P, Medina JL, Henriquez JA, Mishra N, Wagner J, Tokarz R, Cullen JM, Iadarola MJ, Rice CM, Lipkin WI, Kapoor A (2012). Serology-enabled discovery of genetically diverse hepaciviruses in a new host. J Virol.

[2] Kapoor A, Simmonds P, Gerold G, Qaisar N, Jain K, Henriquez JA, Firth C, Hirschberg DL, Rice CM, Shields S, Lipkin WI (2011). Characterization of a canine homolog of hepatitis C virus. Proc Natl Acad Sci U S A.

[3] Global Burden of Disease Study C (2015). Global, regional, and national incidence, prevalence, and years lived with disability for 301 acute and chronic diseases and injuries in 188 countries, 1990–2013: a systematic analysis for the Global Burden of Disease Study 2013. Lancet.

[4] Hoofnagle JH (2002). Course and outcome of hepatitis C. Hepatology.

[5] Scheel TK, Simmonds P, Kapoor A (2015). Surveying the global virome: identification and characterization of HCV-related animal hepaciviruses. Antiviral Res.

[6] Lyons S, Kapoor A, Schneider BS, Wolfe ND, Culshaw G, Corcoran B, Durham AE, Burden F, McGorum BC, Simmonds P (2014). Viraemic frequencies and seroprevalence of non-primate hepacivirus and equine pegiviruses in horses and other mammalian species. J Gen Virol.

[7] Lyons S, Kapoor A, Sharp C, Schneider BS, Wolfe ND, Culshaw G, Corcoran B, McGorum BC, Simmonds P (2012). Nonprimate hepaciviruses in domestic horses, United Kingdom. Emerg Infect Dis.

[8] Drexler JF, Corman VM, Muller MA, Lukashev AN, Gmyl A, Coutard B, Adam A, Ritz D, Leijten LM, van Riel D, Kallies R, Klose SM, Gloza-Rausch F, Binger T, Annan A, Adu-Sarkodie Y, Oppong S, Bourgarel M, Rupp D, Hoffmann B, Schlegel M, Kummerer BM, Kruger DH, Schmidt-Chanasit J, Setien AA, Cottontail VM, Hemachudha T, Wacharapluesadee S, Osterrieder K, Bartenschlager R (2013). Evidence for novel hepaciviruses in rodents. PLoS Pathog.

[9] Tanaka T, Kasai H, Yamashita A, Okuyama-Dobashi K, Yasumoto J, Maekawa S, Enomoto N, Okamoto T, Matsuura Y, Morimatsu M, Manabe N, Ochiai K, Yamashita K, Moriishi K (2014). Hallmarks of hepatitis C virus in equine hepacivirus. J Virol.

[10] Gemaque BS, de Souza JSA, do Carmo Pereira Soares M, Malheiros AP, Silva AL, Alves MM, Gomes-Gouvea MS, Pinho JR, de Ferreira Figueiredo H, Ribeiro DB, Souza da Silva J, Moraes LA, Ribeiro AS, Pereira WL (2014). Hepacivirus infection in domestic horses, Brazil, 2011–2013. Emerg Infect Dis.

[11] Pfaender S, Cavalleri JM, Walter S, Doerrbecker J, Campana B, Brown RJ, Burbelo PD, Postel A, Hahn K, Anggakusuma Riebesehl N, Baumgartner W, Becher P, Heim MH, Pietschmann T, Feige K, Steinmann E (2015). Clinical course of infection and viral tissue tropism of hepatitis C virus-like nonprimate hepaciviruses in horses. Hepatology.

[12] Ramsay JD, Evanoff R, Wilkinson TE, Divers TJ, Knowles DP, Mealey RH (2015). Experimental transmission of equine hepacivirus in horses as a model for hepatitis C virus. Hepatology.

[13] Reuter G, Maza N, Pankovics P, Boros A (2014). Non-primate hepacivirus infection with apparent hepatitis in a horse—Short communication. Acta Vet Hung.

[14] Pfaender S, Brown RJ, Pietschmann T, Steinmann E (2014). Natural reservoirs for homologs of hepatitis C virus. Emerg Microbes Infect.

[15] Gather T, Walter S, Todt D, Pfaender S, Brown RJP, Postel A, Becher P, Moritz A, Hansmann F, Baumgaertner W, Feige K, Steinmann E, Cavalleri JMV (2016) Vertical transmission of hepatitis C virus-like nonprimate hepacivirus in horses. J Gen Virol, in press 10.1099/jgv.0.000561 27461949

[16] Matsuu A, Hobo S, Ando K, Sanekata T, Sato F, Endo Y, Amaya T, Osaki T, Horie M, Masatani T, Ozawa M, Tsukiyama-Kohara K (2015). Genetic and serological surveillance for non-primate hepacivirus in horses in Japan. Vet Microbiol.

[17] Perkins GA, Wagner B (2015). The development of equine immunity: current knowledge on immunology in the young horse. Equine Vet J.

